# Supplementary material for: The Lipid Status in Patients with Ulcerative Colitis: Sphingolipids are Disease-Dependent Regulated
Source: J Clin Med. 2019 Jul 4;8(7):971. doi: 10.3390/jcm8070971 (PMC6678307; doi:10.3390/jcm8070971)
Supplement: Supplementary file 1 [file jcm-08-00971-s001.pdf]

The lipid status in patients with ulcerative colitis: sphingolipids are disease dependent regulated

Supplement:

Table S1: The Mayo Score. The sum of all criteria are used to group the patients into the following disease stages: Remission: 0–1; mild disease: 2–4; moderate disease: 5–6; severe disease: 7–9.

Components of the Mayo Score

Stool Frequency

0 = Normal

1 = 1–2 stools/day more than normal

2 = 3–4 stools/day more than normal

3 = >4 stools/day more than normal

Rectal bleeding

0 = none

1 = visible blood with stool less than half the time

2 = visible blood with stool half of the time or more

3 = passing blood alone

Physician rating of disease activity

0 = normal

1 = mild

2 = moderate

3 = severe

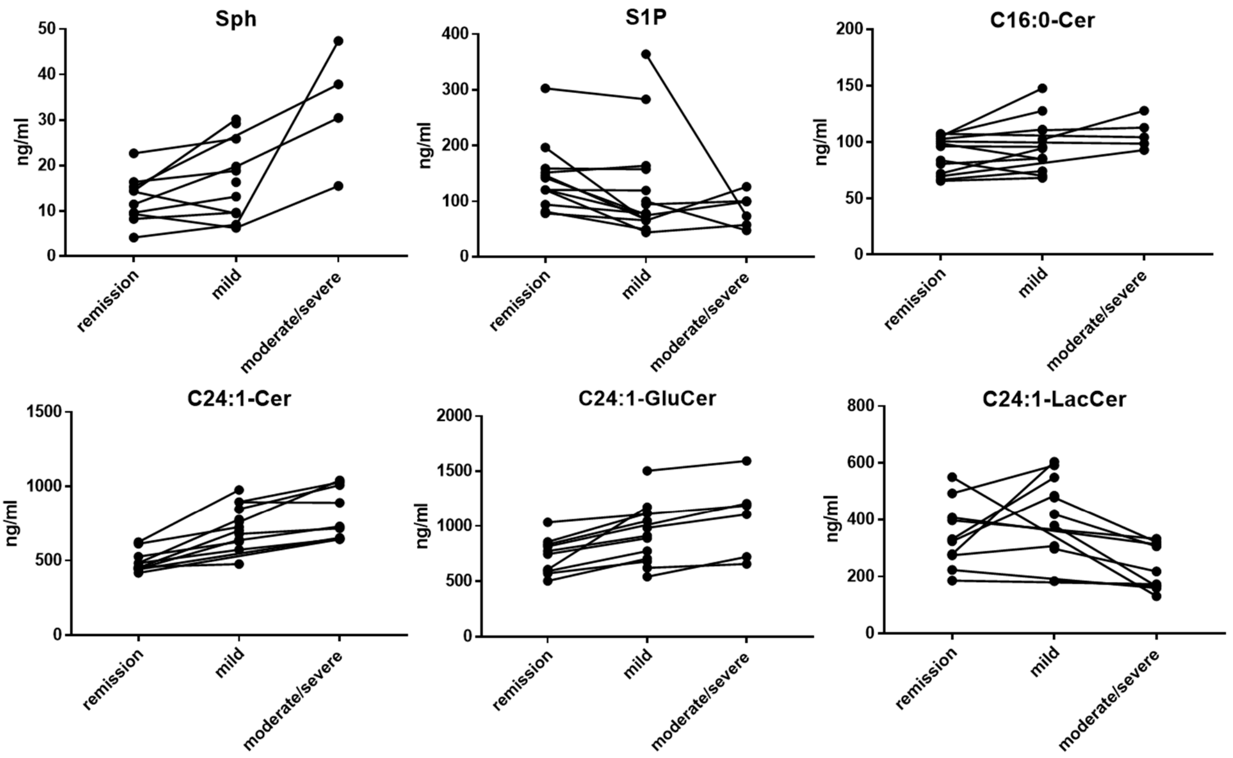

Figure S1. Plasma sphingolipid changes of single patients in dependency of disease stage.

Sphingolipids in plasma samples from single patient who changed their disease stages within the followed up time of this study where analysed by LC-MS/MS and shown as before-after blot. Point to point lines represent changes within one patient.

A

	SPH d18:0	S1P d18:1	S1P d18:0	GlcCer d18:1/16:0	LacCer d18:1/16:00	LacCer d18:1/24:0	LacCer d18:1/24:1	GlcCer d18:1/24:1	GlcCer d18:1/18:0	GlcCer d18:1/18:1	LacCer d18:1/18:0	Cer d18:1/24:0	Cer d18:1/16:0	Cer d18:1/14:0	Cer d18:1/18:0	Cer d18:1/20:0	Cer d18:1/18:1	Cer d18:1/24:1	Cer d18:0/16:0	Cer d18:0/18:0	Cer d18:0/24:0	Cer d18:0/24:1
SPH d18:1 Pearson r	0.697	-0.092	-0.001	0.330	-0.074	0.370	0.162	0.384	0.156	0.245	0.191	0.182	0.286	0.313	0.182	0.404	0.351	0.145	0.166	0.085	0.070	0.071
SPH d18:1 P-value	0.000	0.111	0.985	0.000	0.196	0.000	0.005	0.000	0.006	0.000	0.001	0.001	0.000	0.000	0.023	0.000	0.000	0.011	0.038	0.294	0.383	0.378
SPH d18:0 Pearson r		-0.009	0.107	0.204	-0.030	0.312	0.199	0.235	0.102	0.131	0.180	0.101	0.184	0.227	0.087	0.274	0.161	0.071	0.171	0.046	0.073	0.014
SPH d18:0 P-value		0.876	0.076	0.001	0.615	0.000	0.001	0.000	0.089	0.030	0.003	0.091	0.002	0.008	0.313	0.000	0.062	0.239	0.047	0.594	0.401	0.873
S1P d18:1 Pearson r			0.739	-0.141	0.126	-0.116	-0.024	-0.262	-0.055	-0.084	-0.009	-0.151	-0.172	0.020	-0.056	-0.320	0.072	-0.080	-0.159	-0.099	-0.114	-0.101
S1P d18:1 P-value			0.000	0.012	0.025	0.044	0.675	0.000	0.326	0.137	0.871	0.007	0.002	0.796	0.471	0.000	0.379	0.156	0.039	0.199	0.140	0.189
S1P d18:0 Pearson r				-0.071	0.104	-0.013	-0.009	-0.165	-0.012	-0.007	0.025	-0.046	-0.079	0.087	-0.008	-0.146	0.088	-0.035	-0.035	-0.019	-0.019	-0.020
S1P d18:0 P-value				0.208	0.067	0.825	0.872	0.003	0.837	0.907	0.656	0.418	0.161	0.263	0.922	0.009	0.281	0.531	0.652	0.805	0.806	0.793
GlcCer d18:1/16:0 Pearson r					0.486	0.371	0.208	0.714	0.672	0.644	0.510	0.508	0.710	0.553	0.561	0.652	0.515	0.449	0.355	0.424	0.312	0.394
GlcCer d18:1/16:0 P-value					0.000	0.000	0.000	0.000	0.000	0.000	0.000	0.000	0.000	0.000	0.000	0.000	0.000	0.000	0.000	0.000	0.000	0.000
LacCer d18:1/16:0 Pearson r						0.202	0.459	0.288	0.471	0.420	0.491	0.208	0.314	0.403	0.282	0.107	0.310	0.244	0.239	0.167	0.223	0.293
LacCer d18:1/16:0 P-value						0.000	0.000	0.000	0.000	0.000	0.000	0.000	0.000	0.000	0.000	0.059	0.000	0.000	0.002	0.031	0.004	0.000
LacCer d18:1/24:0 Pearson r							0.372	0.333	0.352	0.303	0.527	0.314	0.356	0.368	0.213	0.488	0.311	-0.064	0.344	0.137	0.260	0.111
LacCer d18:1/24:0 P-value							0.000	0.000	0.000	0.000	0.000	0.000	0.000	0.000	0.008	0.000	0.000	0.272	0.000	0.092	0.001	0.172
LacCer d18:1/24:1 Pearson r								0.354	0.131	0.298	0.259	0.034	0.106	0.209	0.099	0.090	-0.054	0.050	0.599	0.169	0.020	0.011
LacCer d18:1/24:1 P-value								0.000	0.019	0.000	0.000	0.543	0.059	0.006	0.202	0.108	0.506	0.374	0.000	0.028	0.793	0.882
GlcCer d18:1/24:1 Pearson r									0.492	0.462	0.357	0.213	0.497	0.423	0.322	0.544	0.388	0.439	0.342	0.290	0.078	0.355
GlcCer d18:1/24:1 P-value									0.000	0.000	0.000	0.000	0.000	0.000	0.000	0.000	0.000	0.000	0.000	0.000	0.314	0.000
GlcCer d18:1/18:0 Pearson r										0.521	0.635	0.333	0.469	0.294	0.397	0.417	0.294	0.333	0.105	0.206	0.180	0.329
GlcCer d18:1/18:0 P-value										0.000	0.000	0.000	0.000	0.000	0.000	0.000	0.000	0.000	0.171	0.007	0.019	0.000
GlcCer d18:1/18:1 Pearson r											0.320	0.344	0.465	0.602	0.485	0.451	0.466	0.182	0.459	0.429	0.208	0.293
GlcCer d18:1/18:1 P-value											0.000	0.000	0.000	0.000	0.000	0.000	0.000	0.001	0.000	0.000	0.007	0.000
Pearson rLacCer d18:1/18:0												0.283	0.528	0.264	0.480	0.471	0.389	0.355	0.164	0.259	0.210	0.317
LacCer d18:1/18:0 P-value												0.000	0.000	0.001	0.000	0.000	0.000	0.000	0.032	0.001	0.006	0.000
Cerd18:1/24:0 Pearson r													0.550	0.419	0.401	0.545	0.258	0.451	0.332	0.312	0.738	0.334
Cerd18:1/24:0 P-value													0.000	0.000	0.000	0.000	0.001	0.000	0.000	0.000	0.000	0.000
Cerd18:1/16:0 Pearson r														0.693	0.804	0.778	0.762	0.516	0.454	0.623	0.364	0.471
Cerd18:1/16:0 P-value														0.000	0.000	0.000	0.000	0.000	0.000	0.000	0.000	0.000
Cerd18:1/14:0 Pearson r															0.576	0.556	0.743	0.210	0.487	0.430	0.344	0.277
Cerd18:1/14:0 P-value															0.000	0.000	0.000	0.006	0.000	0.000	0.000	0.000
Cerd18:1/18:0 Pearson r																0.868	0.690	0.528	0.447	0.762	0.271	0.436
Cerd18:1/18:0 P-value																0.000	0.000	0.000	0.000	0.000	0.000	0.000
Cerd18:1/20:0 Pearson r																	0.580	0.457	0.457	0.673	0.388	0.465
Cerd18:1/20:0 P-value																	0.000	0.000	0.000	0.000	0.000	0.000
Cerd18:1/18:1 Pearson r																		0.352	0.299	0.494	0.191	0.273
Cerd18:1/18:1 P-value																		0.000	0.000	0.000	0.018	0.001
Cerd18:1/24:1 Pearson r																			0.061	0.265	0.248	0.484
Cerd18:1/24:1 P-value																			0.428	0.000	0.001	0.000
Cerd18:0/16:0 Pearson r																				0.591	0.422	0.376
Cerd18:0/16:0 P-value																				0.000	0.000	0.000
Cerd18:0/18:0 Pearson r																					0.412	0.637
Cerd18:0/18:0 P-value																					0.000	0.000
Cerd18:0/24:0 Pearson r																						0.600
Cerd18:0/24:0 P-value																						0.000

B

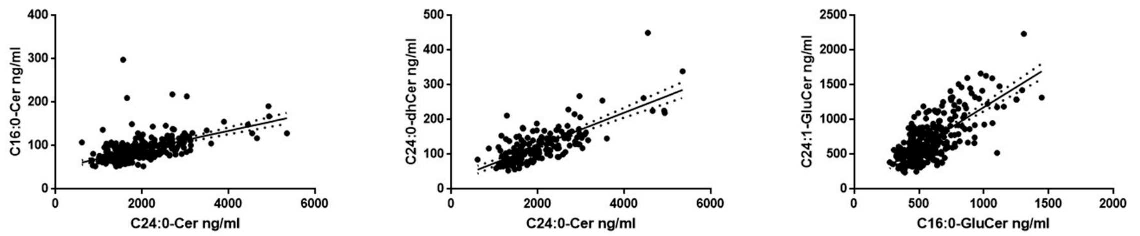

Figure S2: Correlation analysis of plasma sphingolipid level

Plasma sphingolipid level from all UC patients were correlated with each other. Correlation was done with GraphPad Prism7, using Pearson correlation coefficients. P values are shown for each correlation coefficient. Strong ($r > 0.5$) to very strong ($r > 0.7$) positive correlations are highlighted in yellow.
